# TLR4-mediated activation of the ERK pathway following UVA irradiation contributes to increased cytokine and MMP expression in senescent human dermal fibroblasts

**DOI:** 10.1371/journal.pone.0202323

**Published:** 2018-08-17

**Authors:** Seong-Wook Seo, Seul-Ki Park, Soo-Jin Oh, Ok Sarah Shin

**Affiliations:** Department of Biomedical Sciences, College of Medicine, Korea University Guro Hospital, Seoul, Republic of Korea; NYU Langone Medical Center, UNITED STATES

## Abstract

Exposure to ultraviolet (UV) radiation is a major contributing factor to premature aging (photoaging) and skin cancer. *In vitro* models of cellular senescence have proven to be very useful for the study of slow and progressive accumulation of damage resulting in the growth arrest of aging skin cells. In this study, we compared UVA-induced cellular responses in non-senescent (NS) vs. senescent (S) human dermal fibroblasts (HDFs). HDFs were irradiated with a single dose of UVA (7.5 J/cm^2^) and QuantSeq 3' mRNA sequencing was performed to assess differential gene expression. Both NS and S HDFs expressed similar numbers of differentially expressed genes, although distinct sets of genes were differentially expressed between the two groups. Higher expression of matrix metalloproteinases (MMPs) and Toll-like receptor (TLR) pathway genes, such as *TLR4*, *MyD88*, and *CXCL-8*, was detected in S HDFs as compared with NS HDFs, and UVA exposure led to a downregulation of collagen genes, such as *COL8A2* and *COL5A3*. Consistent with gene expression profiling, enhanced IL-6 and IL-8 secretion was observed in S HDFs compared with NS HDFs, in response to UVA. Furthermore, we show that TLR4-mediated ERK pathway is responsible for the UVA-mediated mitochondrial dysfunction as well as increased secretion of MMP-1 and IL-8 in S HDFs. Taken together, our results demonstrate the UVA-induced common and distinct molecular patterns of cellular responses between NS and S HDFs and suggest TLR4/ERK pathways as candidate targets to reduce senescent phenotypes.

## Introduction

Ultraviolet (UV) irradiation causes premature skin aging, called photoaging, characterized by suppressed collagen gene expression and increased expression of matrix metalloproteinases (MMPs), potentially leading to skin cancer [1, 2]. Dramatic alterations in the appearance of human skin with aging are due to the progressive weakening of the delicate framework of the connective tissue components of the dermis [3]. The UV spectrum can be divided into three distinct wavelength ranges: UVA (320–400 nm), UVB (290–320 nm), and UVC (<290 nm). While UVB mainly acts on the epidermal basal layer of the skin, inducing DNA damage and defective repair, UVA penetrates through the epidermis into the dermis, leading to connective tissue damage as a result of degradation of the structural components of the extracellular matrix [4, 5].

Normal somatic cells undergo only a limited number of cellular doublings in culture before irreversibly arresting proliferation, a process known as cellular replicative senescence. Senescent cells generally display a distinct enlarged and flattened morphology with phenotypic changes, including resistance to apoptosis and altered gene expression patterns [6]. Recent studies have illustrated that senescence induces an upregulation of genes that encode secreted proteins with pro-inflammatory properties, including cytokines and chemokines, as well as various growth factors and proteases that together alter tissue structure and function [7]. A phenomenon called senescence-associated secretory phenotype (SASP) is a known characteristic of senescent cells, which involves secretion of proteins, such as interleukin-(IL)-8, IL-1, insulin-like growth factor, and other soluble factors [8]. Thus, blocking these molecules can be potentially therapeutic for aging-related diseases [9].

Given that UVA is considered less carcinogenic than UVB radiation, which directly causes DNA damage, most previous studies have focused on the molecular and immunological changes induced by UVB rather than UVA. However, recent studies have highlighted the importance of UVA in the development of photoaging because UVA is the most abundant source of solar UV exposure and penetrates deeper into the dermis as compared to UVB [3, 5, 10]. The underlying mechanism by which UVA mediates cellular change in an aging skin cell model remains to be fully understood.

Here, we studied the mechanisms of UVA-induced cellular responses in both non-senescent (NS) and senescent (S) human dermal fibroblasts (HDFs). Following UVA irradiation in HDFs, QuantSeq 3' mRNA sequencing was performed to comprehensively measure host gene expression, providing a global view of UVA-specific mRNA profiles. Furthermore, our data demonstrated that Toll-like receptor 4(TLR4)-mediated activation of the ERK pathway contributes to the increased expression of MMP-1 and IL-8 as well as mitochondrial dysfunction, potentially leading to skin photoaging in S HDFs.

## Materials and methods

### Cell culture and senescence model

Human dermal fibroblasts (HDFs) were purchased from Lonza (Basel, Switzerland). For replication-induced senescence, HDFs were grown as adherent cultures in fibroblast basal medium supplemented with fibroblast growth medium (FGM) SingleQuots (Lonza). A model of replicative senescence was developed as previously described [11, 12]. Briefly, HDFs were allowed to grow for more than 20 passages, and S HDFs were verified by their delayed population-doubling times and by using a Senescence β-Galactosidase Staining Kit (Cell Signaling Technology, MA, USA). After staining, the cells were washed and assessed by light microscopy (Olympus, Tokyo, Japan). NS HDFs were considered early-passage number cells (less than 7 passages with negative SA-β-gal staining), whereas S HDFs were considered late-passage number cells [more than 20 passages with positive senescence-associated β-galactosidase (SA-β-gal) staining].

### UV irradiation

UV irradiation was performed as previously described [13]. Briefly, UV irradiation was performed in a BioSUN system illuminator from VL (Vilber Lourmat, France). Cells were washed with phosphate buffered saline (PBS) and irradiated in culture medium with UVA supplied with T-8.L 8W-365 nm tubes or UVB supplied with 8W-312 nm tubes. The cell viability at different time points was determined by 3-[4,5-dimethylthiazol-2-yl]-2,5 diphenyl tetrazolium bromide (MTT) assay.

### Drug treatment

Prior to UV irradiation, cells were pre-treated with various concentrations of the ERK inhibitor U0126 (Sigma-Aldrich, MO, USA) or TLR4 inhibitor TAK242 (Cayman Chemicals, MI, USA). Immediately before irradiation, the culture medium was removed and cells were washed twice in PBS. After UVA exposure, the cells were cultured in fresh media and incubated for an additional amount of time before being collected for further analysis.

### Reactive oxygen species (ROS) assay

Intracellular production of ROS was measured as previously reported [14]. For ROS measurements, cells were exposed to the various UVA doses in pre-warmed PBS. After exposure, cells were stained in PBS containing 5 μM of the oxidation-sensitive fluorescent probe dye, 2',7'-dichlorofluorescein diacetate (DCF-DA; Sigma-Aldrich) for 15 min at 37°C. After staining, cells were again incubated for 15 min at 37°C in pre-warmed PBS. Fluorescence was recorded using a spectrofluorometer (VICTOR3, Perkin-Elmer, MA, USA) at an excitation wavelength of 490 nm and an emission wavelength of 525 nm.

### Immunofluorescence staining

Cells were grown on cover slips and irradiated with UV. At the indicated time points, cells were fixed in 4% paraformaldehyde and used for immunostaining. First, the cells were permeabilized with 0.25% Triton X-100 in PBS for 10 min and washed with PBS, and then subsequently incubated with primary antibodies against phospho-specific γH2AX (Cell Signaling) antibody overnight at 4°C. Next day, the cover slips containing the cells were washed with PBS and incubated with the Alexa 594-conjugated secondary antibodies (Invitrogen) diluted in 0.5% bovine serum albumin (BSA) in PBS/0.05% Tween for 1 h at 37°C in the dark. Cover slips were wet-mounted onto slides using mounting media containing 4,6-diamidino-2-phenylindole (DAPI) and fluorescent images from multiple fields of view were captured using a confocal microscope (LSM700; Carl Zeiss). γH2AX-foci number per cell was counted for total 100 cells in each experiment and represented in the graph. Mitochondrial superoxide levels were determined by staining with MitoSOX Red (Invitrogen), whereas mitochondrial volume was assessed by staining with MitoTracker Green (Invitrogen).

### Gene expression analysis using QuantSeq 3' mRNA sequencing

NS and S HDFs were irradiated with UVA (7.5 J/cm^2^) and 24 h later, RNA was isolated. Total RNA was isolated using TRIzol reagent (Invitrogen, CA, USA). Preliminary RNA samples were evaluated with the Agilent Bioanalyzer using the Nano RNA Kit. Only samples with RNA integrity number (RIN) > 7.0 were used, and cDNA libraries for each of these samples were generated from 500 ng of total RNA using the QuantSeq 3′ mRNA-Seq Library Prep kit for Illumina (Lexogen) following the manufacturer’s instructions [15]. QuantSeq generates highly strand-specific next-generation sequencing libraries close to the 3′ end of polyadenylated RNA. Briefly, the first cDNA strand is generated through reverse transcription initiated by oligo dT priming. Synthesis of the second cDNA strand is performed by random priming, in a manner that DNA polymerase is efficiently stopped when reaching the next hybridized random primer, so only the fragment closest to the 3′ end gets extended until the end and gets both the adapter sequences necessary for polymerase chain reaction amplification.

### Identification of differentially expressed genes (DEGs) and functional analysis (GO, KEGG)

Genes with ≥ 2-fold change and false discovery rate-adjusted q-value < 0.05 were considered significantly differentially expressed. Further analyses were performed as previously described [16]. The representation of functional groups in each sample relative to the whole genome was investigated using the Expression Analysis Systematic Explorer (EASE) tool within DAVID. The EASE tool uses a modified Fisher’s exact test to measure enrichment of gene ontology (GO) terms. In order to identify enriched GO terms, functionally clustered genes were filtered using an EASE value less than 0.05 and selected. KEGG pathway analysis was performed to determine the most differentially regulated pathways, and KEGG pathways were selected as significantly regulated if the corrected p-values were lesser than 0.05.

### Mitochondrial flux analysis

Measuring energy metabolism in HDFs were previously described [17]. HDFs were plated on XF-96 plates (Seahorse BioSciences, Billerica, MA, USA) at a density of 3.5 x 10^3^ cells/well. On the day of mitochondrial flux analysis, media was changed to Krebs-Henseleit buffer at pH 7.4 and incubated at 37°C in a non-CO_2_ incubator for 1 h. All media and injection reagents used in this assay were adjusted to pH 7.4. Using the XFp Extracellular Flux analyzer, oxygen consumption rate (OCR) was measured following drug injection in HDFs. OCR was automatically calculated and recorded by the Seahorse software. Protein concentration of each sample was determined using Pierce 660 nm Protein assay reagent (Thermo Scientific) and was used to normalize respective OCR.

### Western blotting

Western blotting was performed as previously described [18]. Cells were lysed with RIPA buffer (Sigma-Aldrich) at the specified time points post-UV irradiation. Lysate proteins were resolved by sodium dodecyl sulfate-polyacrylamide gel electrophoresis (SDS-PAGE) on 10–12% acrylamide gels. Proteins were transferred onto polyvinylidene difluoride membranes, and blocked with 5% (w/v) skim milk in Tris-buffered saline (0.2 M Tris, 1.36 M NaCl) supplemented with 0.1% (v/v) Tween-20 (TBS-Tw) for 1 h at room temperature. This was followed by overnight incubation with primary antibodies against phospho-ERK/ERK (Cell Signaling Technology) at 4°C. As a loading control, β-tubulin (Abgent, CA, USA) antibody was used. After three washes in TBS/Tw, the membranes were incubated with horseradish peroxidase-conjugated anti-rabbit or anti-mouse IgG secondary antibodies for 1 h at 25°C. Membranes were then washed with TBS-Tw and incubated with Western Lumi Pico solution (ECL solution kit) (DoGen, Seoul, Korea). A representative image of three independent experiments is shown.

### Enzyme-linked immunosorbent assay (ELISA)

IL-6, IL-8, and MMP-1 ELISA kits were from R&D Systems (Minneapolis, MI, USA). The assay was performed according to the manufacturer’s instructions. Absorbance at 450 nm was measured using a microplate spectrophotometer.

### Statistical analysis

Quantitative data were expressed as mean ± standard error of the mean (SEM). Statistically significant differences between the control and test groups were evaluated by the Student’s *t*-test (Graphpad Prism) and p-values < 0.05 were considered significant.

## Results

### Distinct cellular responses in HDFs following UV exposure

**Fig 1:**
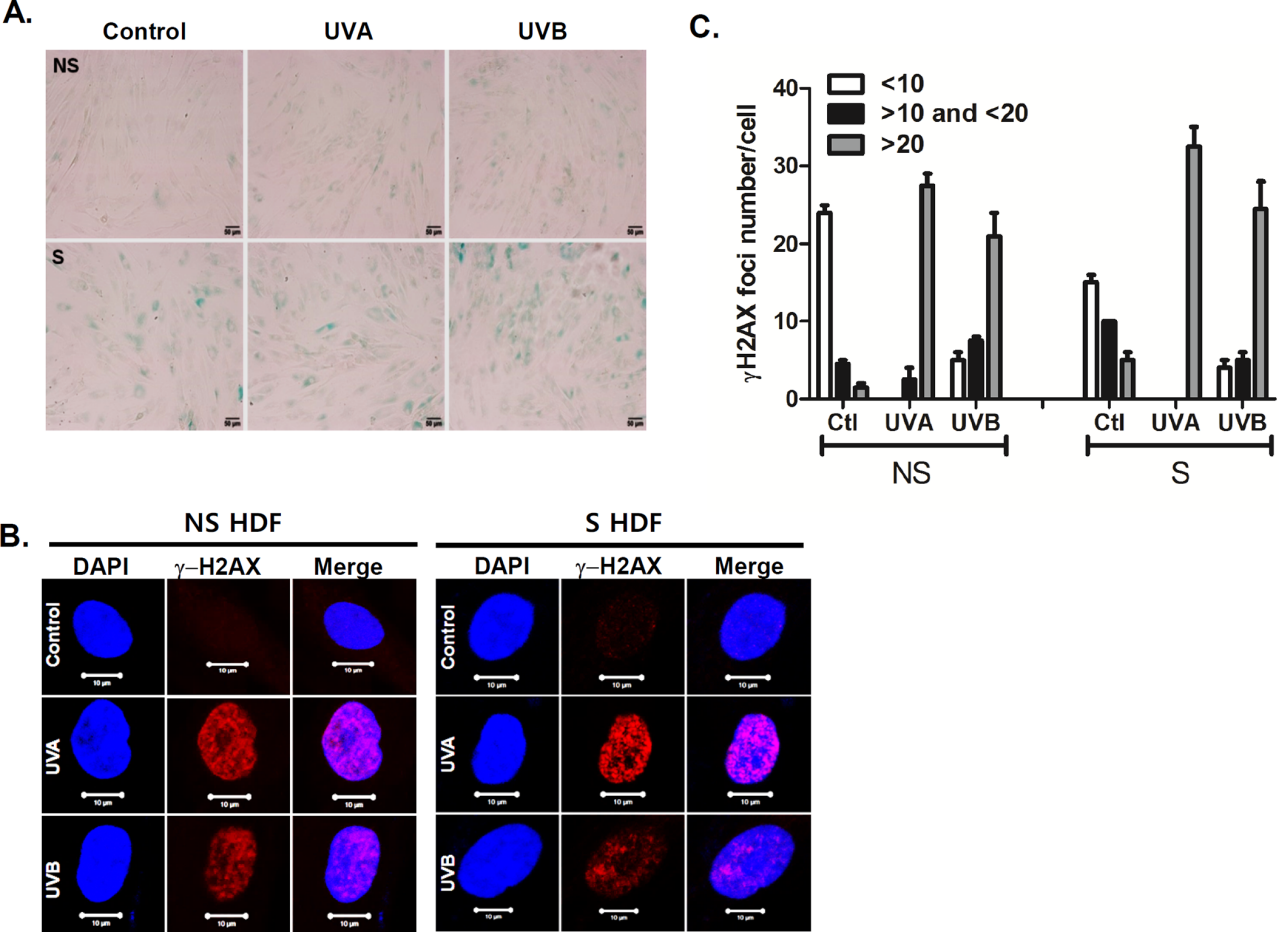
Cellular characteristics of NS and S HDFs following UV exposure. (A)Normal or non-senescent (NS) and senescent (S) HDFs were irradiated with UVA (7.5 J/cm^2^) or UVB (0.25 J/cm^2^). The extent of senescence-associated β-galactosidase (SA-β-Gal) staining after UV irradiation was observed. Scale bar = 50 μm. (B, C) Immunofluorescence analysis of γ-H2AX foci formation was performed by confocal microscopy. DAPI (blue), γ-H2AX foci (red), and merge images are shown. Scale bar = 10 μm. The graph shows the number of γ-H2AX foci per cell. The data represent the mean ± SEM of three independent sets of experiments.

The effects of UVA or UVB irradiation on cell viability were determined by the MTT assay. Following UVA (0.1–20 J/cm^2^) or UVB (0.001–1 J/cm^2^) exposure for 24 h, we observed a dose-dependent decrease in cell viability with UVA (starting at 10 J/cm^2^) and UVB (starting at 0.5 J/cm^2^) (data not shown). Therefore, we chose a dose of 1 or 7.5 J/cm^2^ for UVA and 0.25 J/cm^2^ for UVB, which showed minimal reduction in cell viability, to perform further experiments. To determine the effect of UVA and UVB irradiation on cellular responses in S HDFs, we used late-passage S HDFs according to a previously established aging cell model [11, 12]. Replicative senescence was first described by Hayflick and Moorhead in human fibroblasts, after they observed cells undergoing extensive replication as a consequence of serial culture passages [19]. Given that increased SA-β-gal activity is a well-established biomarker of senescence [20], the primary HDFs were passaged up to more than 20 times until they displayed a senescent cell-like phenotype, and the SA-β-gal positive cells were then counted to validate senescence. NS HDFs were normal cells and S HDFs were cells positive for SA-β-gal staining. As seen in Fig 1A, even without UV irradiation, replication-induced senescence in HDFs resulted in an increase in the number of cells positive for SA-β-gal, as compared to NS HDFs. When cells were exposed to UVA (7.5 J/cm^2^) or UVB (0.25 J/cm^2^) for 24 h, S HDFs showed an increased number of SA-β-gal positive cells as compared to control-treated cells, whereas NS HDFs showed negative staining for SA-β-gal activity.

UVA and UVB irradiations are known to induce DNA double-strand breaks and the formation of DNA damage foci containing activated γH2AX represents DNA damage, which activates the DNA damage response [21]. NS and S HDFs were irradiated with UVA (7.5 J/cm^2^) or UVB (0.25 J/cm^2^). As shown in Fig 1B and 1C, NS and S HDFs were positively stained for γH2AX and the percentage of positively stained cells (>20 γH2AX foci cells) were significantly increased upon UVA or UVB treatment in both groups. Taken together, these results suggest that UVA and UVB can induce DNA damage by enhancing γH2AX foci formation, regardless of the senescence levels of HDFs.

**Fig 2:**
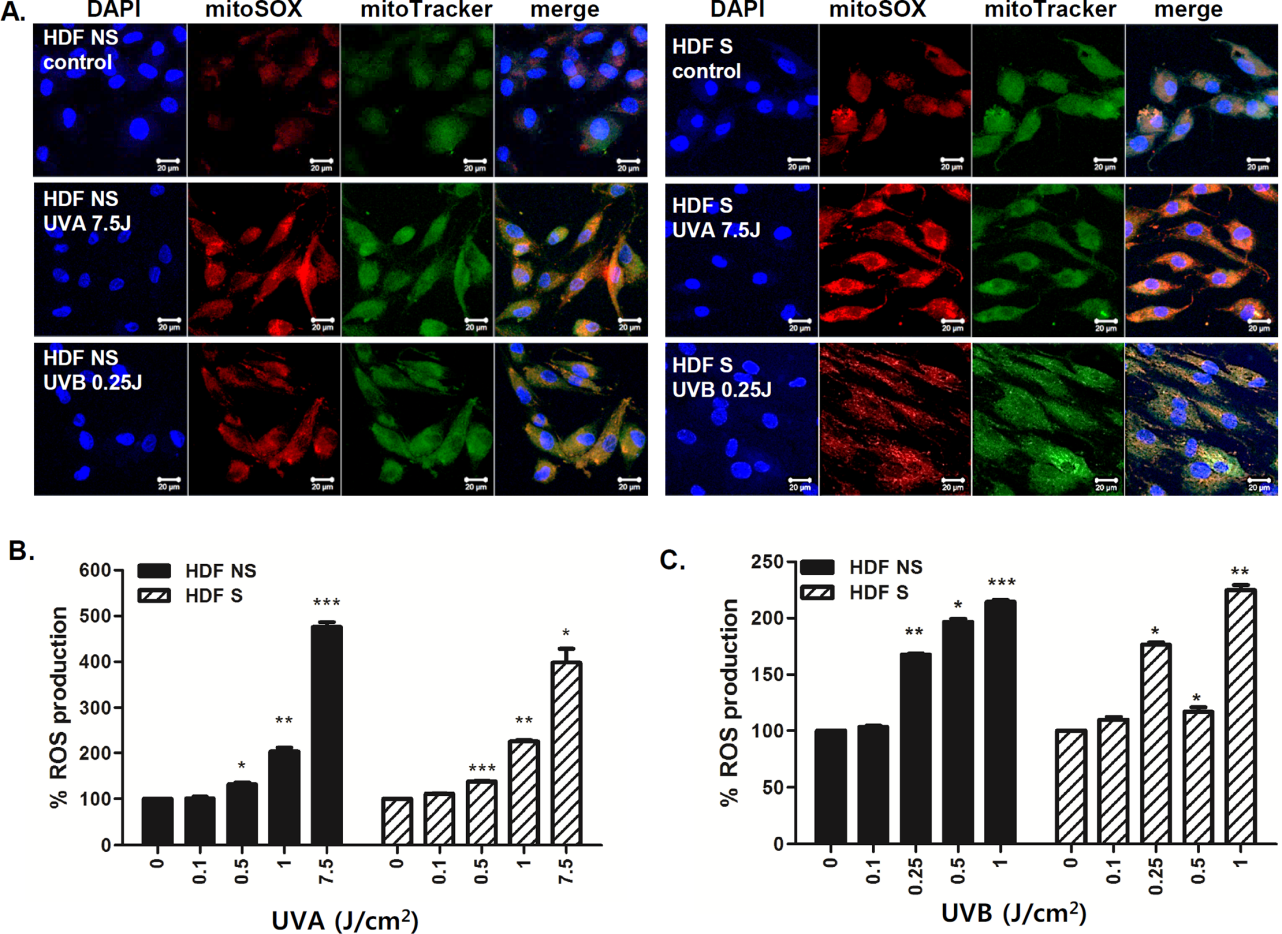
The effect of UV irradiation on ROS production in NS and S HDFs. NS and S HDFs were irradiated with UVA (7.5 J/cm^2^) or UVB (0.25 J/cm^2^) for 2 h. (A) Cells were double-stained with DAPI (blue) and MitoSOX (red) or MitoTracker (green) and examined by confocal microscopy. Images are representative of three independent experiments; scale bar = 20 μm. (B,C) Cells were irradiated with various doses of UVA or UVB for 24 h. Total cellular ROS levels were determined by measuring DCF-DA levels using a spectrofluorometer. Percentage of ROS production in response to UVA (B) and UVB (C) is shown in the graphs. Data are presented as percentage normalized to the control (DMSO-treated cells). *p < 0.05; **p < 0.01; ***p < 0.001 vs. control cells.

It is well established that cellular aging can cause cumulative damage to the mitochondria and to the mitochondrial DNA as a result of ROS production and thus accumulation of ROS-mediated oxidative damage is a key characteristic of aging and senescence [22]. The probe, MitoTracker Green, was used to monitor the mitochondrial volume, whereas MitoSOX Red was used to measure the production of the mitochondrial superoxide anion. High numbers of both NS and S HDFs displayed increased mitochondrial volume as well as mitochondrial superoxide formation in response to UV (Fig 2A). Furthermore, we also tested whether exposure to different doses of UVA or UVB modulates ROS production. UVA (7.5 J/cm^2^) irradiation led to a 5-fold increase in ROS production (Fig 2B), whereas UVB (1 J/cm^2^) irradiation led to a 2-fold increase in ROS production, in both NS and S HDFs (Fig 2C).

### Comprehensive analysis of functional enrichment and pathways of UVA-irradiated NS and S HDFs

**Fig 3:**
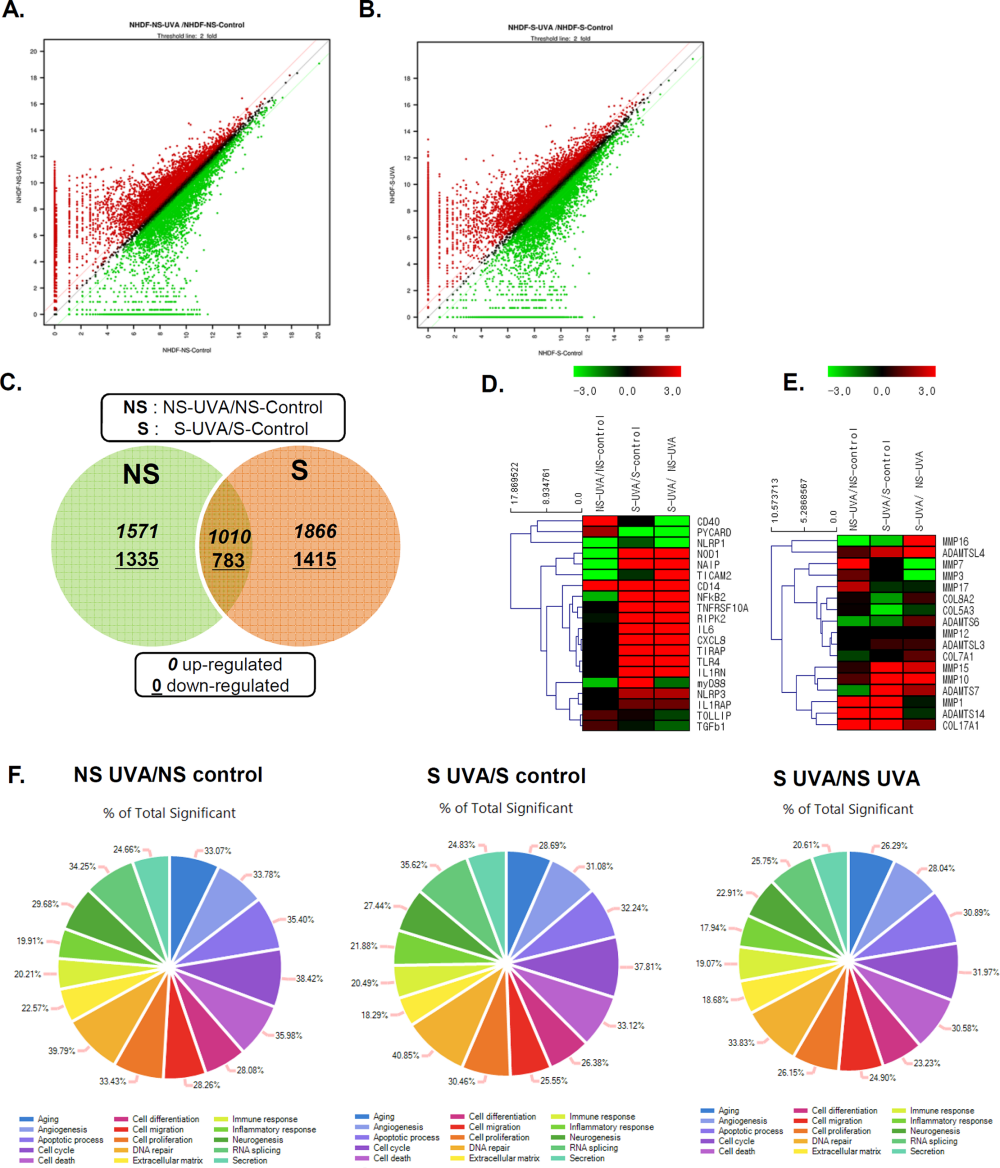
Global overview of the gene expression profile of UVA-irradiated HDFs. NS and S HDFs were irradiated with UVA (7.5 J/cm^2^) for 24 h. RNA was isolated and QuantSeq 3′ mRNA sequencing was performed. (A, B) A scatter plot is shown. The X-axis shows the expression level of genes from control groups, whereas the Y-axis shows the expression level of genes from UVA-irradiated NS (A) and S (B) HDFs. The red dots represent relatively highly expressed genes in UVA-irradiated groups, whereas the green dots represent relatively highly expressed genes in control groups. (C) Venn diagrams of overlapping differentially expressed genes (DEGs) profiles for NS and S cells. DEGs correspond to those displaying a change of more than 2-fold with a q-value of less than or equal to 0.05. The mRNA differential expression levels in UVA-irradiated NS and S cells compared with control are depicted in three overlapping circles for 2-fold up- and downregulation. The numbers indicate the mRNA counts in the indicated area. (D, E) Heatmap displays the enrichment of immune response and inflammation-related genes (D) and extracellular matrix genes (E), such as MMPs, in samples. Red, black, and green colors indicate levels of gene expression above, equal to, and below the mean, respectively. (F) Composite images of gene ontology (GO) graph are shown. The circle and bar indicate the GO terms related to gene functions and percentage of differentially expressed upregulated genes in each category, respectively.

QuantSeq technology has been used as an alternative to microarrays and conventional RNA-seq, in order to measure gene expression [15]. To compare the cellular responses of NS and S HDFs to UVA irradiation, QuantSeq 3′ mRNA sequencing was performed to analyze the transcriptome of UVA-irradiated primary fibroblasts. NS or S HDFs were irradiated with UVA (7.5 J/cm^2^), and RNA was isolated 24 h later. DEGs were identified as genes up- or downregulated with a fold change of ± 2 and a q-value < 0.05, compared to the control (no UVA irradiation). As summarized by expression plots and Venn diagrams, we identified 1571 and 1866 UVA-specific upregulated DEGs in NS and S HDFs, respectively. Moreover, Venn diagrams revealed that 1010 genes were commonly upregulated, whereas 783 genes were commonly downregulated in UVA-irradiated NS and S HDFs (Fig 3A, 3B and 3C).

Some significantly up- or downregulated gene expression profiles determined using heatmap cluster analysis are shown in Fig 3D and 3E. Chemokines, such as chemokine (C-X-C motif) ligand 8 (*CXCL8*), and inflammatory molecules, such as *IL-6*, were among the top 50 most upregulated genes in S HDFs, but their expression levels were found to be unchanged or downregulated in NS HDFs. Interestingly, genes involved in TLR signaling pathways, including *TLR4*, *CD14*, *MyD88*, *RIPK2*, and *NF-κB2* were also found to be highly regulated DEGs in S HDFs (Table 1 and Fig 3D). UVA exposure is shown to induce the synthesis and expression of MMP-1 in dermal fibroblasts, enhancing the degradation of dermal collagen [23, 24]. As shown in Fig 3E, MMP-1 expression was found to be highly upregulated in both NS and S HDFs in response to UVA, whereas the expression of other MMPs varied. Further, the expression levels of the collagen genes, *COL8A2* and *COL5A3*, were found to be downregulated in S HDFs, but remained unchanged in NS HDFs in response to UVA.

**Table 1:** Top 20 up-regulated DEGs of immune function.

	Top 20 up-regulated DEGs (immune function)	NS-UVA/NS-control	S-UVA/S-control	S-UVA/ NS-UVA
1	CD40	292.312	1.000	.003
2	CD14	11.454	370.630	32.358
3	PYCARD	3.749	0.002	.0005
4	IL6	1.000	52.693	52.693
5	NOD1	0.006	95.933	95.933
6	NFκB2	0.231	174.029	174.029
7	TNFRSF10A	1.000	240.412	240.412
8	CXCL8	1.000	31.127	31.127
9	NAIP	0.007	34.427	34.427
10	TIRAP	1.000	33.007	33.007
11	MyD88	0.211	81.958	.408
12	RIPK2	0.856	432.104	432.104
13	TICAM2	0.008	0.674	113.950
14	TLR4	1	76.474	76.474
15	NLRP1	0.015	0.495	0.002
16	NLRP3	1	4.223	4.223
17	IL1RN	1	108.452	108.452
18	IL1RAP	1	2.433	2.433
19	TOLLIP	2.004	1.169	.583
20	TGFβ1	1.852	0.855	.46

**Fig 4:**
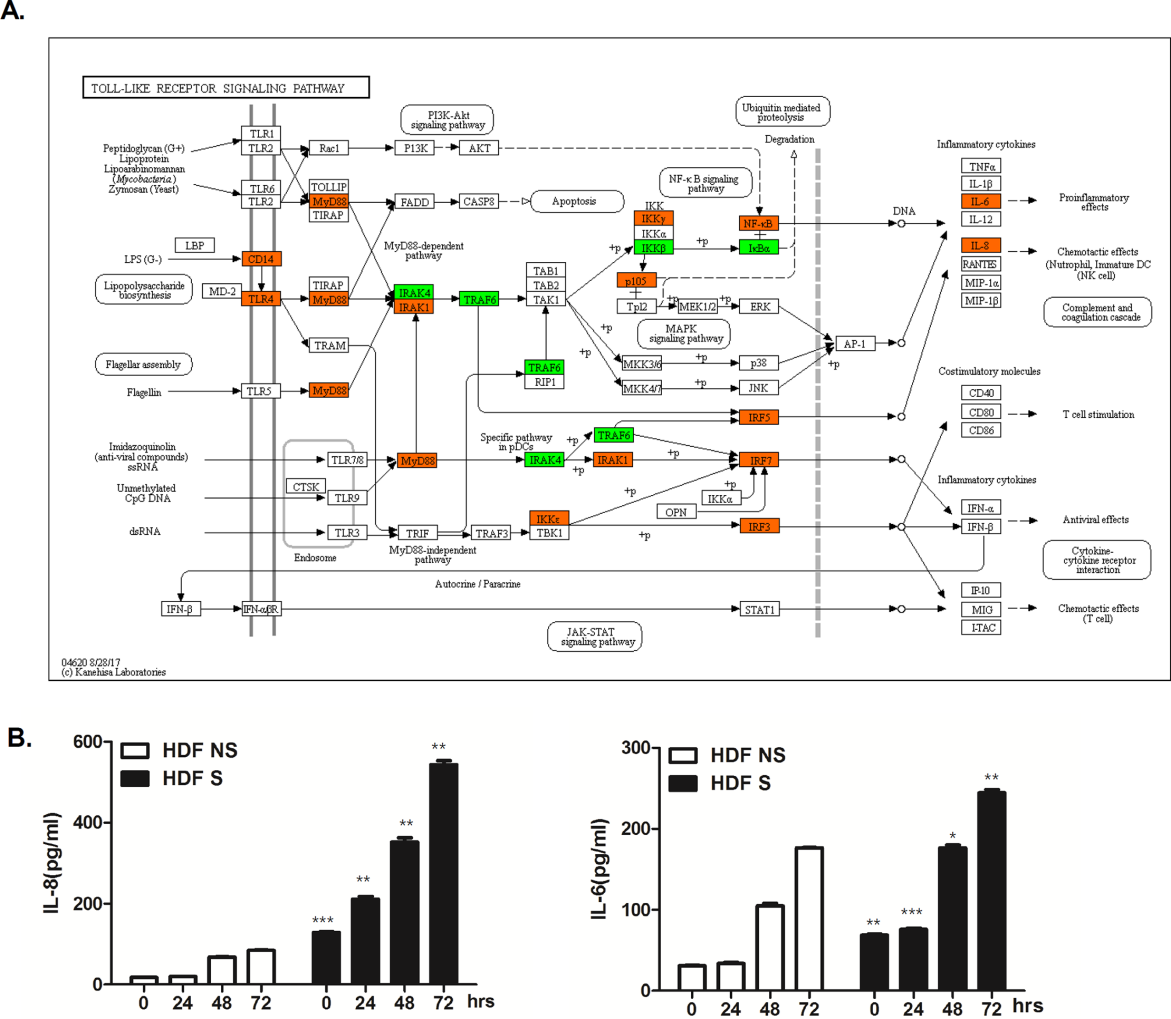
Validation of DEGs in UVA-irradiated S cells that are involved in skin senescence. (A) KEGG analysis shows the list of DEGs involved in Toll-like receptor signaling pathway. The orange, green, and white colors indicate significantly increased, significantly decreased, and unchanged gene expression between normal vs UVA-treated S HDFs, respectively. (B) NS and S HDFs were irradiated with UVA (1 J/cm^2^) and cultured for the indicated time periods (0, 4, 24, 48, and 72 h). IL-6 and IL-8 production levels in the culture media were measured by ELISA. The results are expressed as mean ± SEM of three different experiments. *p < 0.05, **p < 0.01, ***p < 0.001 vs. NS HDFs at each timepoints.

Gene ontology (GO) enrichment analysis revealed that 20.21% of total DEGs were functionally enriched for immune response in NS HDFs, whereas 20.49% of total DEGs were functionally categorized for immune response in S HDFs (Fig 3F). Many DEGs were found to be in functional groups of DNA damage, apoptotic process, cell cycle, and cell death. Higher numbers of DEGs were involved in inflammation in S HDFs than in NS HDFs (21.88% vs. 19.91%). KEGG pathway enrichment analysis showed that these DEGs induced by UVA in S HDFs were found to be associated with the TLR signaling pathway. Upregulated genes are shown in orange and downregulated genes are shown in green. Genes involved in TLR signaling cascades, such as *CD14*, *TLR4*, *MyD88*, *NF-κB*, *IL-6* and *IL-8*, were shown to be upregulated upon UVA irradiation in S HDFs (Fig 4A).

To validate the QuantSeq results, we measured IL-8 and IL-6 secretion levels in the supernatants of UV-irradiated HDFs by ELISA. Following UVA irradiation, we observed increased IL-8 and IL-6 secretion in a time-dependent manner (Fig 4B). Consistent with QuantSeq results, higher levels of IL-6 and IL-8 were secreted in S HDFs irradiated with UVA as compared to NS HDFs. These findings demonstrate a differential response to UVA in NS and S HDFs.

### Inhibition of TLR4 or ERK pathway decreases UVA-induced MMP-1 and IL-8 secretion

**Fig 5:**
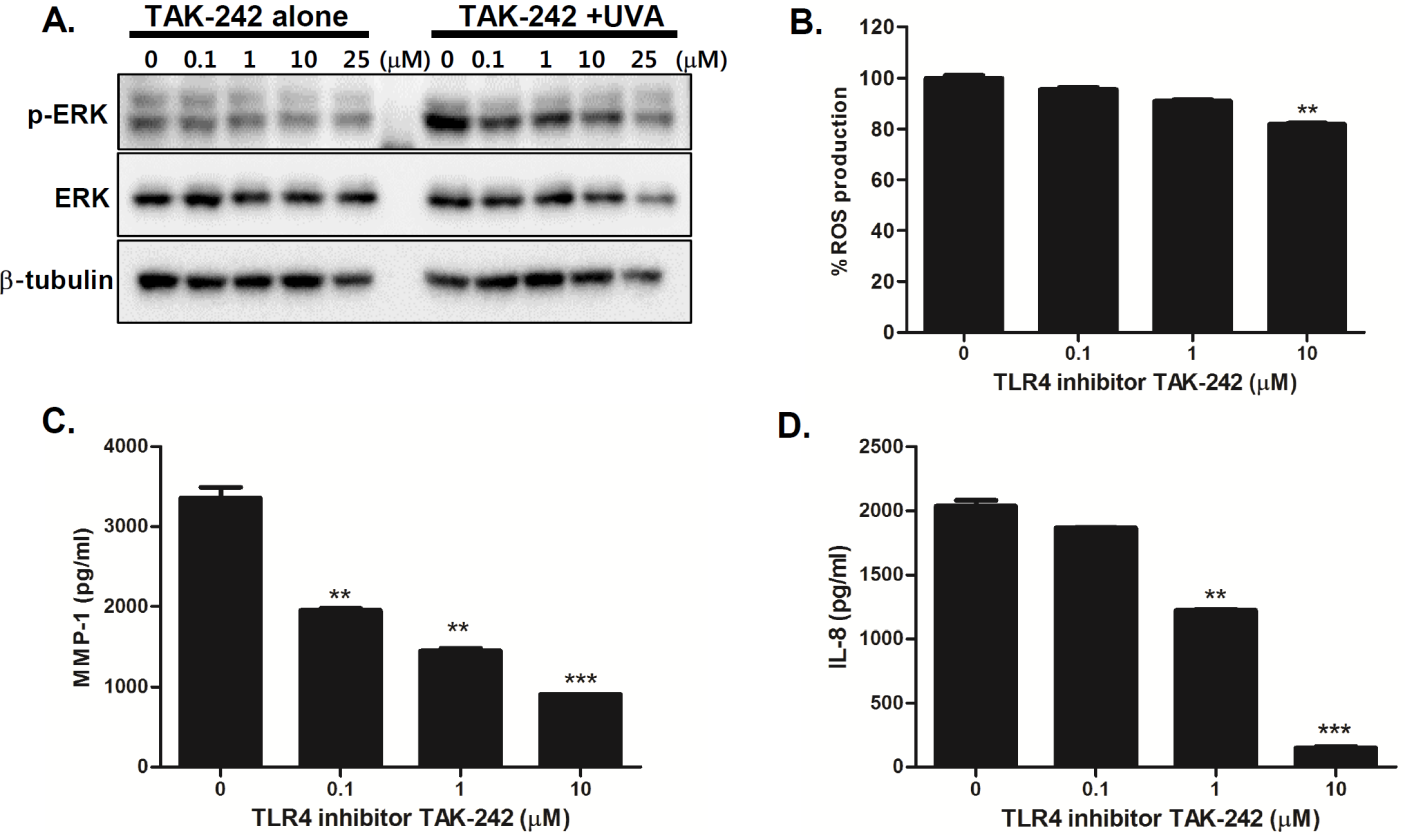
TLR4 inhibition suppresses UVA-induced MMP-1 and IL-8 production in UVA-irradiated senescent HDFs. (A) Senescent HDFs were treated overnight with different concentrations of TAK242 (TLR4 inhibitor) and irradiated with UVA (1 J/cm^2^). Cells were cultured for 4 h and protein levels of phosphorylated/total ERK were analyzed by western blotting. Anti-β-tubulin monoclonal antibody was used as a loading control. The images shown are representative of three independent experiments. (B) Total cellular ROS levels were determined by measuring DCF-DA levels using a spectrofluorometer. % ROS production in response to UVA is shown in the graph. Data are presented as percentage compared to the control (DMSO-treated cells). *p < 0.05, **p < 0.01, ***p < 0.001 vs. control cells. (C) MMP-1 and (D) IL-8 secretion levels in the culture supernatant were measured by ELISA. The values are mean ± SEM of three different experiments. *p < 0.05, **p < 0.01, ***p < 0.001 vs. control cells.

**Fig 6:**
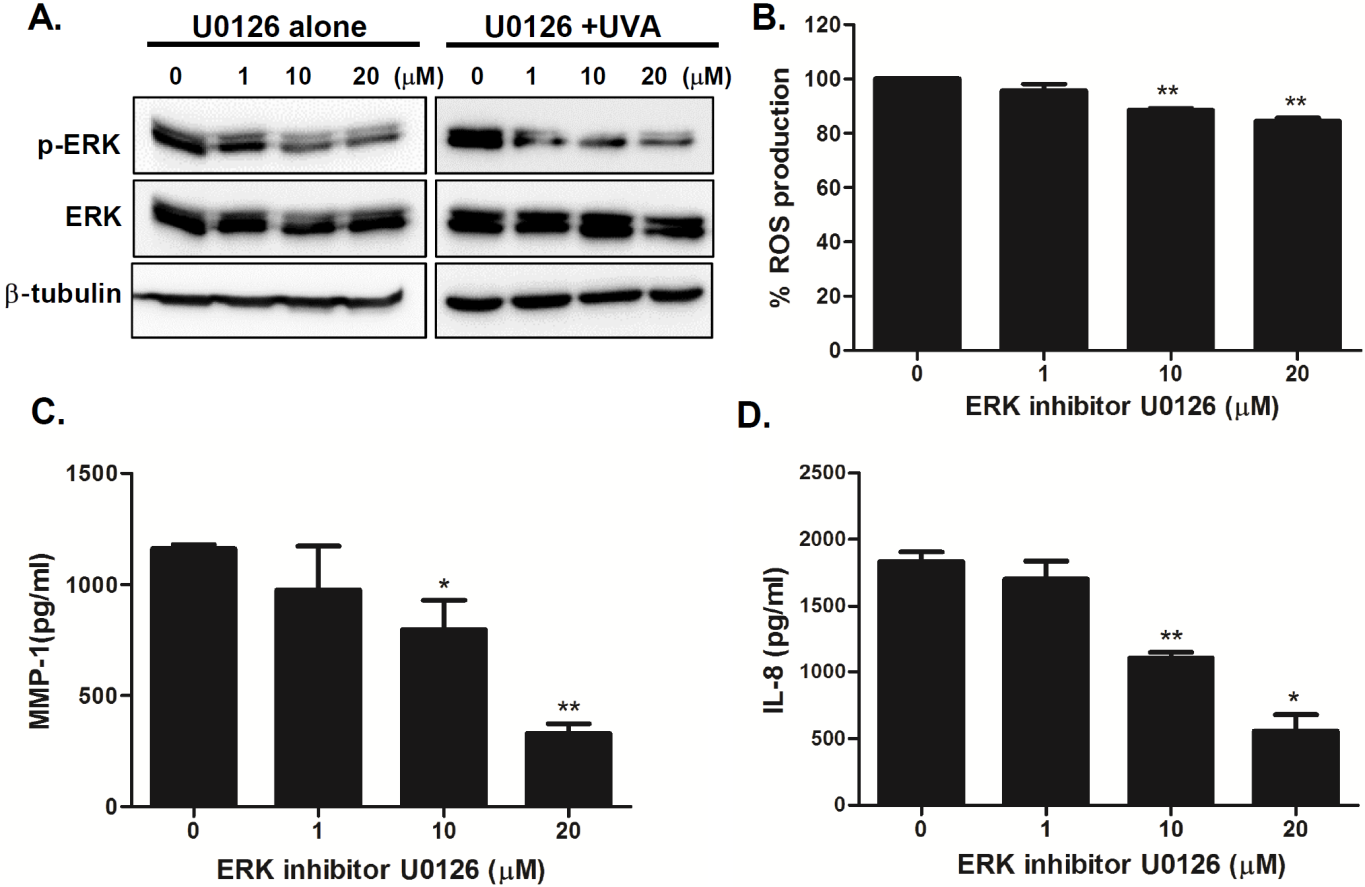
Inhibition of ERK pathway modulates UVA-induced MMP-1 and IL-8 production in UVA-irradiated senescent HDFs. (A) Senescent HDFs were treated overnight with different concentrations of U0126 (ERK inhibitor) and irradiated with UVA (1 J/cm^2^). Cells were cultured for 4 h and protein levels of phosphorylated/total ERK were analyzed by western blotting. Anti-β-tubulin monoclonal antibody was used as a loading control. The images shown are representative of three independent experiments. (B) Total cellular ROS levels were determined by measuring DCF-DA levels using a spectrofluorometer. % ROS production in response to UVA is shown in the graph. Data are presented as percentage compared to the control (DMSO-treated cells). (C) MMP-1 and (D) IL-8 secretion levels in the culture supernatant were measured by ELISA. The values are mean ± SEM of three different experiments. *p < 0.05, **p < 0.01, ***p < 0.001 vs. control cells.

TLR4 signaling is known to trigger the mitogen activated protein kinase (MAPK) and nuclear factor-κB (NF-κB) pathways and MAPK pathways are stimulated upon UV to regulate downstream molecules in HDFs [13, 25, 26]. To further investigate the role of TLR4-mediated pathways in aging features of senescent phenotype in UVA-irradiated S HDFs, we treated cells with various concentrations of TLR4 inhibitor TAK-242 prior to UVA irradiation in S HDFs. TLR4 inhibitor suppressed the phosphorylation of ERK in a dose-dependent manner (Fig 5A). Additionally, there was a dose-dependent decrease in ROS, MMP-1, and IL-8 production, suggesting that the TLR4 pathway is important for UVA-mediated cellular responses in dermal fibroblasts (Fig 5B, 5C and 5D). Similar results were observed with the inhibition of ERK pathway. To investigate whether the inhibition of the ERK pathway would modulate UVA-induced responses, we treated S HDFs with various concentrations of the ERK inhibitor (U0126). The phosphorylation of ERK was downregulated upon ERK inhibitor treatment in a dose-dependent manner (Fig 6A). Furthermore, ERK inhibitor pretreatment led to suppression of ROS, MMP-1, and IL-8 production, suggesting that the ERK pathway is important for UVA-mediated cellular responses in S HDFs (Fig 6B, 6C and 6D).

**Fig 7:**
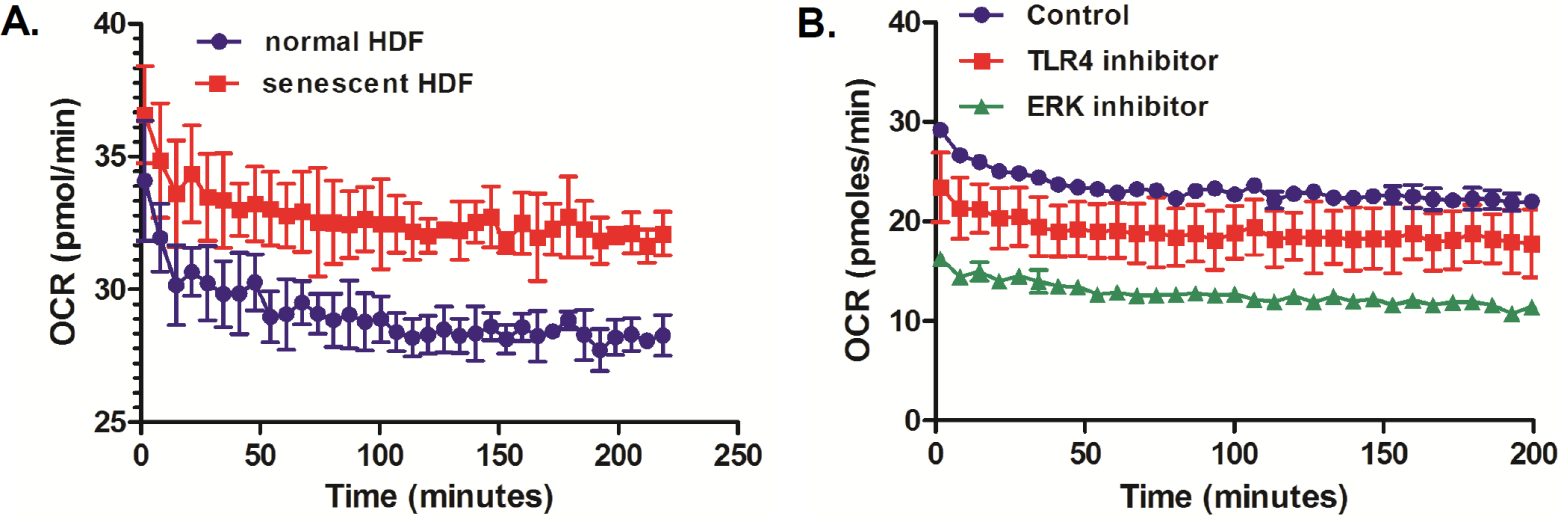
TLR4 or ERK-specific inhibitor treatment reduces oxygen consumption rate (OCR) in UVA-irradiated senescent HDFs. (A) OCR was measured in normal (NS) and senescent (S) HDFs over 3 hours. (B) The graph shows changes in OCR in control, TLR4 inhibitor (10μM)-treated, ERK inhibitor (10μM) treated UVA-irradiated senescent HDFs. The results are expressed as mean ± SEM of three biological replicates.

One of key features for cellular senescence is aging-dependent mitochondrial degeneration [27]. To evaluate whether ERK or TLR4 inhibition would result in change in mitochondrial function of senescent HDFs, we used a Seahorse XF analyzer to measure the metabolic changes in mitochondrial respiration in a realtime measurement of OCR. Previous reports suggested a hyperfunctional mitochondrial in senescent fibroblasts [28]. Similar to this finding, our results also indicate that senescent HDFs exhibited higher OCR numbers than non-senescent (normal) HDFs (Fig 7A). Furthermore, ERK or TLR4 inhibitor-treated senescent HDFs had strikingly decreased OCR than control-treated cells, suggesting that TLR4-ERK signaling pathways can lead to control of mitochondrial metabolic profilings in senescent HDFs (Fig 7B)

## Discussion

Numerous studies have focused on UVB-mediated skin damage, whereas less attention has been paid to UVA-mediated responses. UVA penetrates deeper into the dermis than UVB and is known to induce ROS production, and alters the expression of dermal collagen and MMPs [3, 4, 10]. Our data highlight the distinct and dynamic changes in the expression patterns of host genes following UVA irradiation in NS and S HDFs. A better understanding of the global gene expression changes underlying the multi-step progression of UVA-induced skin damage could help develop potential therapeutic strategies for photoaging and skin cancer.

Given that cellular senescence is correlated with a general change in transcriptome [29] including higher expression of senescence-associated secretory phenotype (SASP) genes, we compared the transcriptomic changes in NS and S HDFs in response to UVA stimulation. We observed that UVA exposure led to a higher expression levels of the pro-inflammatory molecules (IL-6 or IL-8) in S HDFs than in NS HDFs. This finding correlates well with the KEGG pathway analysis presented in this study, in which significantly upregulated DEGs were mostly associated with TLR4 and chemokine signaling pathways. Thus, it is highly possible that as compared with NS HDFs, expression of higher levels of inflammatory molecules from S HDFs in response to UVA will contribute to accelerating skin aging.

Following UVA irradiation, activation of the immune system through pathways involving TLRs demonstrates an important regulatory function of TLR pathways in skin immunity and aging. Several studies have suggested that TLR2, 3, 4, 7, 8, and 9 may be involved in DNA repair mechanisms and UVB-induced apoptosis [30, 31]. Furthermore, a recent study by Bald *et al*. demonstrated that UV radiation promotes melanoma progression via recruitment and activation of neutrophils, initiated by the production of high mobility group box 1 from UV-stimulated keratinocytes, and this inflammation is driven by TLR4 signaling pathway [32]. In line with these findings, our results also indicate that the expression of multiple genes involved in TLR4 signaling pathway is significantly increased upon UVA irradiation in S HDFs. Altered TLR function or single nucleotide polymorphisms within TLRs have been implicated in various human diseases, hence TLRs are being considered as potential therapeutic targets to treat various diseases [33]. Furthermore, there exists a close association between TLRs and various skin pathologies and diseases, including atopic dermatitis, psoriasis, and melanoma and non-melanoma skin cancers [26]. Thus, it will be interesting to include further studies to test the effect of TLR antagonists in aging-related diseases.

It is generally believed that mitogen-activated protein kinase (MAPK) can be activated downstream of TLR signaling, and that the components of MAPK signaling pathway are targets of UVA irradiation and are important for the regulation of UVA-induced cellular responses in several different cell types [23, 25, 34, 35]. Our data show that UVA can induce the phosphorylation of ERK pathway, and that inhibition of pERK can lead to suppressed production of MMP-1 and IL-8 in HDFs. Another important finding from our study is that TLR4 or ERK inhibition can lead to suppression of mitochondrial OCR production. Mitochondrial metabolism control is known to be essential for aging features of senescence and thus may act as a potential target for blocking senescent phenotype [27, 36]. Real-time measurements of mitochondrial respiration profiling are highly likely to reflect the functional bioenergetic capacity of mitochondria [37]. Further detailed studies on the role of TLR4 and ERK pathways on the control of mitochondrial dynamics and the mechanisms underlying ROS generation will be interesting to follow. Moreover, a photoprotective effect of TLR4 or ERK inhibitor can be further studied by evaluation of these drugs using *in vivo* aging animal model.

Premature skin aging in response to UV exposure is probably due to qualitative and quantitative changes in the dermal extracellular matrix, resulting in increased fragility and impaired wound healing. MMPs are a family of zinc-requiring proteases with a broad range of substrate targets and are largely responsible for the degradation of various extracellular matrix proteins, such as collagen [38]. Among MMP family members, MMP-1 is the most well characterized family member, which causes degradation of type I collagen and associated with connective tissue remodeling and decreases collagen production following UV-induced skin damage. Our heatmap data shows that MMP-1 expression levels were similarly increased in both NS and S HDFs in response to UVA. However, other MMPs such as MMP3 or MMP7 were downregulated in S HDFs, whereas these MMPs were upregulated in NS HDFs. Taken together, this data suggests specific MMP expression may be associated with aging skin cell phenotypes.

In summary, we characterized a comprehensive transcriptome analysis of UVA-specific responses and demonstrated that several biological pathways related to TLR and inflammation pathways are associated with highly upregulated DEGs. Differential expression of specific proteins involved in the TLR4/ERK pathway may explain the severity of skin aging by replicative senescence. These findings provide a framework for further studies examining the molecular mechanisms underlying skin aging and senescence.

## Supporting information

S1 File [QuantSeq Report_2018.xlsx]: The list of DEGs from NS vs. S HDFs in response to UVA. The file includes the data underlying DEGs from QuantSeq 3′ mRNA sequencing.
(XLSX)
